# Prospective Study of Leptospirosis Transmission in an Urban Slum Community: Role of Poor Environment in Repeated Exposures to the *Leptospira* Agent

**DOI:** 10.1371/journal.pntd.0002927

**Published:** 2014-05-29

**Authors:** Ridalva D. M. Felzemburgh, Guilherme S. Ribeiro, Federico Costa, Renato B. Reis, José E. Hagan, Astrid X. T. O. Melendez, Deborah Fraga, Francisco S. Santana, Sharif Mohr, Balbino L. dos Santos, Adriano Q. Silva, Andréia C. Santos, Romy R. Ravines, Wagner S. Tassinari, Marília S. Carvalho, Mitermayer G. Reis, Albert I. Ko

**Affiliations:** 1 Centro de Pesquisas Gonçalo Moniz, Fundação Oswaldo Cruz, Ministério da Saúde, Salvador, Brazil; 2 Escola de Enfermagem, Universidade Federal da Bahia, Salvador, Brazil; 3 Instituto de Saúde Coletiva, Universidade Federal da Bahia, Salvador, Brazil; 4 Department of Epidemiology of Microbial Diseases, School of Public Health, Yale University, New Haven, Connecticut, United States of America; 5 Secretaria Estadual de Saúde da Bahia, Salvador, Brazil; 6 Escola Nacional da Saúde Pública, Fundação Oswaldo Cruz, Ministério da Saúde, Rio de Janeiro, Brazil; 7 Universidade Federal Rural do Rio de Janeiro, Rio de Janeiro, Brazil; David Geffen School of Medicine at UCLA, United States of America

## Abstract

**Background:**

Leptospirosis has emerged as an urban health problem as slum settlements have rapidly spread worldwide and created conditions for rat-borne transmission. Prospective studies have not been performed to determine the disease burden, identify risk factors for infection and provide information needed to guide interventions in these marginalized communities.

**Methodology/Principal Findings:**

We enrolled and followed a cohort of 2,003 residents from a slum community in the city of Salvador, Brazil. Baseline and one-year serosurveys were performed to identify primary and secondary *Leptospira* infections, defined as respectively, seroconversion and four-fold rise in microscopic agglutination titers. We used multinomial logistic regression models to evaluate risk exposures for acquiring primary and secondary infection. A total of 51 *Leptospira* infections were identified among 1,585 (79%) participants who completed the one-year follow-up protocol. The crude infection rate was 37.8 per 1,000 person-years. The secondary infection rate was 2.3 times higher than that of primary infection rate (71.7 and 31.1 infections per 1,000 person-years, respectively). Male gender (OR 2.88; 95% CI 1.40–5.91) and lower per capita household income (OR 0.54; 95% CI, 0.30–0.98 for an increase of $1 per person per day) were independent risk factors for primary infection. In contrast, the 15–34 year age group (OR 10.82, 95% CI 1.38–85.08), and proximity of residence to an open sewer (OR 0.95; 0.91–0.99 for an increase of 1 m distance) were significant risk factors for secondary infection.

**Conclusions/Significance:**

This study found that slum residents had high risk (>3% per year) for acquiring a *Leptospira* infection. Re-infection is a frequent event and occurs in regions of slum settlements that are in proximity to open sewers. Effective prevention of leptospirosis will therefore require interventions that address the infrastructure deficiencies that contribute to repeated exposures among slum inhabitants.

## Author Summary

Leptospirosis is a disease that is transmitted by human contact with an environment contaminated with urine from animals, such as rodents, infected by the *Leptospira* bacteria. Human illness due to these bacteria can be mild, or can have very severe complications. Residents of urban slum settlements are at high risk for this disease, but the specific risk factors for transmission in these settlements are not understood because of the lack of prospective studies in this epidemiological setting. We performed a prospective study in a Brazilian slum community to measure the risk of infection, identify the environmental and social factors that place slum residents at risk for infection, and determine whether some individuals are at risk of repeated infections. We identified a burden of infection with leptospirosis among slum residents, and found that male gender and low income both increase the risk for infection. In addition, a significant proportion of slum residents had a second exposure to leptospirosis and re-infection occurred most frequently among young adults and the poorest members of the slum community who reside in proximity of open sewers. These risk factors are amenable to interventions aimed to reduce the burden that leptospirosis imparts in this high-risk setting.

## Introduction

Leptospirosis is a bacterial disease that has emerged as a major health problem in the developing world [1]. The disease is caused by a spirochete from the genus *Leptospira*, which colonizes the kidney of a wide range of mammals [2]. Human infection occurs after direct contact with an infected animal reservoir, or water and soil contaminated with their urine [1].Infection produces a broad spectrum of clinical manifestations, which may lead from an asymptomatic and mild self-limiting febrile illness to severe disease forms with high case fatality [3]. Leptospirosis has traditionally been a sporadic rural-based disease associated with occupational risk groups such as subsistence farmers [3]. However, changes in human demography during the last 50 years have raised awareness of the emergence of leptospirosis as an urban health problem [4].

Rapid urbanization and urban poverty have led to the dramatic growth of slum settlements throughout low and middle-income countries [5]. To date one billion of the world's population reside in urban slums; this population continues to expand at rates of 10% per year [5]. As a consequence of poor sanitation in these communities, slum residents are increasingly exposed and are at risk of acquiring water and animal-borne diseases [6], [7] such as leptospirosis [1], [4], [8]. In slum settings, endemic transmission of leptospirosis is largely due to circulation of a single serogroup, *L. interrogans* serogroup Icterohaemorrhagiae, [4], [7], [9], [10] for which the domestic rat is the maintenance host [4], [11]. In tropical urban environments, increased transmission and seasonal outbreaks occur during periods of heavy rainfall and flooding [4], [12], [13]. Furthermore extreme climatic events such as monsoons, typhoons and hurricanes have precipitated urban epidemics, as exemplified by the Mumbai outbreak in 2005 [14] and more recently, in the Philippines in 2009 [15] and Australia in 2011 [16].

Moreover, leptospirosis imparts a large disease burden as the cause of life-threatening infection among slum dwellers. In Brazil, more than 10,000 cases of leptospirosis are reported each year [17], the large majority of whom are residents of urban slums and require hospitalization [18] for severe complications of Weil's disease and leptospirosis-associated pulmonary hemorrhage syndrome (LPHS) [1], [7], [19]. Overall case fatality is >10% among reported cases from Brazil [4] and >50–70% for cases that develop LPHS [7], [19]. However, severe disease represents a small fraction of the overall disease burden [20], [21] and to date, prospective studies have not been performed to identify the risk of leptospirosis among slum dwellers.

Investigations of urban leptospirosis, which have used ecological [4], [12], [22], cross-sectional [9] and case-control study designs [23], [24], have identified infrastructure deficiencies in the environment where slum dwellers reside as risk factors for acquiring leptospirosis and anti-*Leptospira* antibodies. For example, high risk of *Leptospira* transmission has been found to be associated with proximity of residence to open sewers and accumulated refuse, flood-risk areas, and areas with high rat infestation [9], [12], [22]–[26]. In addition to environmental features, low socioeconomic status among slum residents contributes to the risk of leptospirosis [12], [22] and anti-*Leptospira* antibodies [9]. However these investigations are limited by the ecological study design, or in the case of cross-sectional surveys, the use of anti-*Leptospira* antibodies, which are detected in individuals up to four or more years after infection [27], [28]. To date, there are no studies that have attempted to evaluate prospectively the risk factors for leptospirosis among urban slum populations.

We previously reported the findings of a large seroprevalence survey [9] of a slum community in Salvador, a city in Northeast Brazil where 33% of the population reside in slum settlements [29] and seasonal rainfall-associated epidemics of leptospirosis occur each year [4], [23]. This study found that a large proportion (15.4%) of slum inhabitants had anti-*Leptospira* antibodies, suggesting that in addition to high rates of infection, repeated exposures with the *Leptospira* agent may be occurring in this high-risk population. Herein, we report findings from a prospective investigation of this urban slum population to determine the risk of *Leptospira* infection and identify risk associations for infection.

## Methods

### Ethics

Participants were enrolled according to written informed consent procedures approved by the Institutional Review Boards of the Oswaldo Cruz Foundation and Brazilian National Commission for Ethics in Research, Brazilian Ministry of Health, Weill Medical College of Cornell University, and Yale School of Public Health.

### Study site and participants

The cohort study was conducted in the Pau da Lima slum, a community situated in the periphery of Salvador (population, 2,675,656 inhabitants) [29], Brazil. The study site has been previously described [9]. Briefly, it comprised a four-valley area of 0.46 Km^2^ with poor sanitation infrastructure. In 2003, the study team performed a census in the study site and identified 14,122 inhabitants residing in 3,689 households. The median household per capita income was US$ 1.30 per day, and most (85%) of the studied population were squatters without legal title to their domiciles. A sample of 684 (18.5%) households from a database of all enumerated households identified within the study site during the 2003 census was selected using a random number generator. Household sampling was chosen to facilitate follow-up evaluations and avoid excluding family members from a part of the study protocol in which other members are participating. The sample size of this sub-cohort was selected to detect a risk ratio of at least 2.0 for exposure risk factors, and was guided by seroprevalence surveys in this community, which identified a seroprevalence of 15% [9], and case-control investigations that determined that the frequency of risk exposures for leptospirosis is between 20–40% in community individuals [23]. All participants who slept three or more nights per week in the sampled households and had five or more years of age were eligible for enrollment in the cohort study.

### Epidemiological data collection

Participants were enrolled between February 2003 and May 2004. In household visits during baseline cohort enrollment, the study team of nurse technicians, physicians and nurses administered a standardized questionnaire to obtain information on demographic and socioeconomic indicators, employment and occupation, exposures to sources of environmental contamination, and presence of potential reservoirs and domestic animals, including rats, chickens, dogs, and cats, in the household and workplace. Information on race was self-reported, and interpreted as a marker of socioeconomic status. The study team evaluated literacy according to the ability to read standardized sentences and interpret their meaning. Informal work was defined as work-related activities for which the participant did not have legal working documents. Frequent exposure to contaminated environment was defined by contact with mud, floodwater, garbage or sewage in the one-month period preceding data collection. Participants were asked to report the highest number of rats sighted within the household property and workplace site in the preceding one-month period. The head-of-household, defined as the member who earned the highest monthly income, was interviewed to determine sources and amounts of income for the household. The study team surveyed the area within <10 meters of the household to determine the presence of vegetation.

Between September and October, 2004, the study team surveyed the study site to record the location of open sewage and rainwater drainage systems. We also mapped the sites of accumulated refuse and measured the area of these deposits. Geographic Information Systems (GIS) was used to obtain three-dimensional distance from the household to the nearest drainage systems and accumulated refuse, and to the lowest point in the valley (height) [9].

### Serologic evaluation

The study team collected blood samples from participants during the baseline survey and a follow-up survey conducted between October 2004 and January 2005. Sera were evaluated using the microscopic agglutination test (MAT) as previously described [9] to determine titers of agglutinating antibodies against a panel of five reference strains (WHO Collaborative Laboratory for Leptospirosis, Royal Tropical Institute, Holland) and two clinical isolates [4] The use of this reduced panel of strains, which represent five *Leptospira* serovars, Autumnalis, Canicola, Copenhageni, Ballum, and Grippotyphosa, demonstrated similar performance during laboratory confirmation of leptospirosis cases [4] and seroprevalence surveys [23], [30] in studies performed in Salvador, Brazil, as the use of the WHO-recommended panel of 16 reference serovars [31]. Screening was performed with serum dilutions of 1∶25, 1∶50 and 1∶100. When agglutination was observed at a dilution of 1∶100, the sample was titrated to determine the highest agglutination titer. The absence and presence of a positive agglutinating antibody titer during the baseline survey was used to differentiate primary and secondary *Leptospira* infections which occurred during follow-up of the cohort. A primary infection was defined as seroconversion during which the MAT titer increased from negative during the baseline survey to a titer ≥1∶50 during the follow-up survey. A secondary infection was defined as a four-fold rise in MAT titer in a participant who had a titer of ≥1∶25 during the baseline survey. The MAT was repeated for samples of participants who were defined as having primary and secondary infections, in order to confirm their status.

### Statistical methods

Epidemiological and laboratory data were double-entered using the Epi-Info for Windows software (Centers for Disease Control and Prevention, Atlanta, GA). There were no missing values for any of the analyzed variables. Data for individual participants were linked by location of residence to spatially coded information for households and environmental attributes within the study site. We used Chi-square and Wilcoxon rank sum tests to compare categorical and continuous data, respectively, between participants who were and were not selected to participate in the cohort, between participants who consented and did not consent to be enrolled in the cohort and between cohort participants who completed and who did not complete the study follow-up. A P-value of 0.05 or less in two sided testing was used as criteria for a statistically significant difference.

We calculated infection rates and 95% confidence intervals according to the Poisson distribution for primary, secondary and overall *Leptospira* infections, adjusting for the design effect of the household-based cluster sampling strategy. Only participants who completed follow up were included in the analysis. Rates were expressed as infections per 1,000 person-years of follow-up.

We applied multinomial logistic regression models in both univariate and multivariate analysis to assess the relationship between explanatory variables and the occurrence of primary and secondary infections as compared to participants without evidence for incident serological infection. Interpretation of results was based on the odds ratio and 95% confidence intervals. Confounding and interaction between independent variables were evaluated by subgroup analyses prior to performing the logistic model. The results obtained in the univariate multinomial logistic regression models were confirmed using binomial logistic regression models, for which the outcome was independently primary infection versus no infection, and secondary infection versus no infection.

Variables that had significant association at a P≤0.10 in the univariate multinomial logistic model were selected to be incorporated into a hierarchical multinomial multivariate model [32] that accounted for hierarchical inter-relationships between variables and the potential underestimation of the effects of distal determinants. The hierarchical model grouped variables into three blocks; the first block contained socioeconomic variables, such as illiteracy, educational attainment, and per capita daily household income. The second block contained household variables, such as number of residents per household, time residing at the same household, household flooding, household distances to the lowest point in valley and to nearest open sewer, presence of vegetation in <10 meters from the household, and presence of potential reservoirs in the household. The third block comprised of the individual-level variables: gender, age, contact with floodwater, sewage water or trash, excavating or cleaning an open sewer, and risk related occupations. A multinomial backward elimination strategy was then performed for each block. Variables that reached a P value ≤0.10 in each of the three blocks were then selected and grouped into a final block. Multinomial backward elimination was pursued on the final block of variables and those reaching a P value ≤0.10 associated with one of the two types of infection were included in the final model. A P value <0.05 was considered statistically significant.

## Results

### Recruitment and follow-up of study participants

Of the 14,122 inhabitants within the study site, 12,651 (90%) were eligible to participate in the cohort and 2,419 (19%) were randomly selected by household for study enrollment. Participants who were selected for cohort recruitment were similar to participants who were not selected in regards to median age (23 versus 24 years, respectively; P: 0.38) and proportion of males (47% versus 48%, respectively; P: 0.18). Among the 2,419 selected participants, 2,003 (83%) consented for the cohort study. Those who agreed to be a cohort member were younger than those who refused to participate (median age in years, 23 vs. 25, P: 0.02) and were less likely to be male (44% versus 61%; P: <0.001).

Of the 2,003 enrolled participants, 1,585 (79%) completed the one-year follow-up study protocol. Participants were followed for a median of 306 days (minimum of 140 and maximum of 657 days). The major reason for loss to follow-up was moving to a household outside the study site (60% of the loss-to-follow-up participants). Participants who completed follow up differed from participants who did not in that they had a lower proportion of males (42% versus 51%; P: 0.002), had a lower educational level (23% completed primary school versus 30%; P: <0.01), and had a lower income (median household monthly income per capita US$ 39 versus 42, respectively, P: 0.03).

### *Leptospira* infection rate

**Figure 1:**
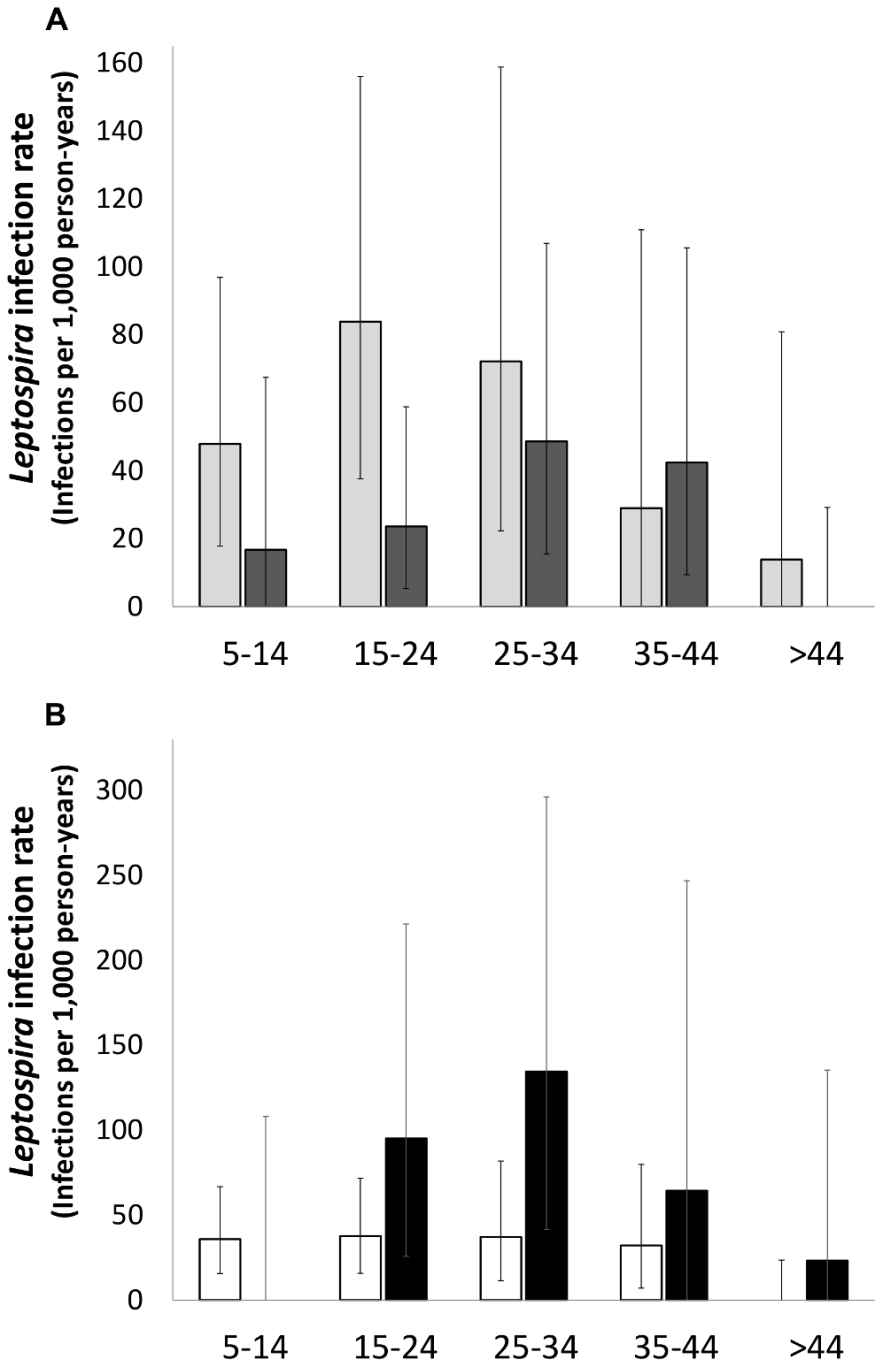
Age-specific attack rates for A) overall *Leptospira* infection according to gender [light grey bars, male; dark grey bars, female], and B) primary and secondary *Leptospira* infection rates [open bars, primary infection; black bars, secondary infection]. Whiskers indicate the 95% Confidence Interval for the rate, adjusted for the survey design. The x axes refer to age groups in years. Rates in y axes are shown as infections per 1,000 person-years

Overall, 51 (3.2%) among the 1,585 participants who completed the follow-up had serological evidence for acquisition of *Leptospira* infection. None of the participants who had *Leptospira* infection reported having been diagnosed with leptospirosis in a health care facility, hospitalized for an acute febrile illness, or identified as a case of leptospirosis during active city-wide hospital-based surveillance for leptospirosis. Highest MAT titres were observed in agglutination reactions against serovars Copenhageni and Autumnalis for samples from 50 (98%) and 1 (2%), respectively, of the 51 individuals with confirmed infection. The overall crude *Leptospira* infection rate was 37.8 infections per 1,000 person-years (95% CI: 26.3–51.9) (Table 1). The infection rate adjusted for age and gender of the eligible population did not significantly differ from the crude infection rates (data not shown). Infection rates were higher among the group with 15 to 24 years of age (47.9 infections per 1,000 person-years; 95% CI: 24.5–81.3) and with 25 to 34 years of age (58.1 infections per 1,000 person-years; 95% CI: 27.4–103.6). Males had 2.12 (95% CI: 1.22–3.69) times greater risk of infection than females (54.5 infections per 1,000 person-years [95% CI: 33.8–81.4] versus 25.6 infections per 1,000 person-years [95% CI: 13.9–41.9], respectively) (Table S1). The gender difference in infection risk was most prominent in the group with 15–24 years of age (RR 3.55, 95% CI: 1.28–9.88, Table S1, Figure 1A).

**Table 1:** *Leptospira* infection rates among 1,585 participants of the slum community cohort according to gender.

Type	Total			Males			Females		
	No. cases	No. PY	Rate^a^ (95% CI)	No. cases	No. PY	Rate^a^ (95% CI)	No. cases	No. PY	Rate^a^ (95% CI)
**All infections**	51	1,349	37.8 (26.3–51.9)	31	569	54.5 (33.8–81.4)	20	780	25.6 (13.9–41.9)
** Primary infection** b	35	1,126	31.1 (19.9–45.4)	22	462	47.6 (26.7–76.2)	13	664	19.6 (8.9–35.6)
** Secondary infection** c	16	223	71.7 (35.8–123.6)	9	107	84.1 (31.2–170.3)	7	116	60.3 (18.7–132.8)

Abbreviations: CI: confidence intervals adjusted according to design effect, PY: person-years of follow-up.

a Rates expressed as infections per 1,000 person-years.

b Primary infection was defined as an increase in the microscopic agglutination test (MAT) titer for any of the tested serovars from zero in the first test to at least 50 in the second test.

c Secondary infection was defined as an increase in the MAT of four-fold from an initial titer ≥25.

During the two-year period from the initiation of cohort enrollment to the end of the follow-up protocol, active hospital-based surveillance in Salvador identified five suspected cases of leptospirosis among the 12,651 inhabitants of the study site who were identified during the baseline census and were eligible to participate in the cohort study. Among suspected cases, four and one had a laboratory-confirmed and probable, respectively, diagnosis of leptospirosis. All cases had highest MAT titres directed against *L. interrogans* serovar Copenhageni. None of the leptospirosis cases was a member of the study cohort. Based on the eligible population at the site, the annual incidence for severe leptospirosis was 19.8 (7.2–43.8) cases per 100,000 population.

### Primary and secondary infection rates

Of the 51 participants who had serological evidence for *Leptospira* infection, 35 had a baseline MAT titer equal to zero (1,126 person-years of follow-up) and 16 had a baseline MAT titer ≥1∶25 (223 person-years of follow-up), which was defined as a marker for a previous infection. The primary and secondary infection rates were 31.1 (95% CI: 19.9–45.4) and 71.7 (95% CI: 35.8–123.6) per 1,000 person-years, respectively. The risk for secondary infections was significantly higher than primary infection during follow-up of participants who were seropositive and seronegative, respectively, at enrollment (RR: 2.31; 95% CI: 1.30–4.10).

The age groups of 15–24 and 25–34 years had the highest secondary infection rates of 95.2 and 134.6 cases per 1,000 person-years, respectively, whereas the primary infection rates in these age groups was 37.7 and 37.2 infections per 1,000 person-years, respectively (p = 0.094 and p = 0.046 respectively, Figure 1B). Although secondary infection rates were similar for males and females (RR: 1.39; 95% CI: 0.54–3.61), primary infection rates were significantly higher among males (RR: 2.43; 95% CI: 1.24–4.78) (Table S1). The gender difference in primary infection rates was greatest for the group with 15–24 years of age; males had a more than four-fold greater risk (RR: 4.22; 95% CI: 1.14–15.59) of acquiring primary infection more than females in this age group.

### Risk factors for *Leptospira* infection

Due to the difference in age and gender-specific rates for primary and secondary infections, the univariate multinomial models found young adults with 15–34 years of age to have significantly increased risk for secondary infection (OR: 8.64; 95% CI: 1.12–66.65), but not for primary infection (Table 2). Male gender was associated with primary infection (OR: 2.37; 95% CI: 1.19–4.74), but not with secondary infection. Socioeconomic variables, including illiteracy, low per capita household income, and lack of a CPF card (financial identification number) were found to be risk factors for primary infection, but not for secondary infection. Each one-dollar increase in the daily per capita household income decreased the odds of primary infection by 50% (95% CI: 0.28–0.89).

**Table 2:** Selected univariate risk factors for *Leptospira* infection among cohort participants.

Risk factor	Uninfected n = 1,534	Primary infection n = 35		Secondary infection n = 16	
	No. or median (% or IQR)	No. or median (% or IQR)	OR (95% CI)	No. or median (% or IQR)	OR (95% CI)
Age group (years)					
5–14	466 (30.4)	13 (37.4)	1.00 (NA)	1 (6.3)	1.00 (NA)
15–34	647 (42.2)	18 (48.6)	0.94 (0.45–1.93)	12 (75.0)	8.64 (1.12–66.65)
≥35	421 (27.4)	5 (14.3)	0.43 (0.15–1.20)	3 (18.8)	3.33 (0.34–32.03)
Male gender	639 (41.7)	22 (62.9)	2.37 (1.19–4.74)	9 (56.3)	1.80 (0.67–4.86)
Illiteracy	259 (16.9)	13 (37.1)	2.80 (1.38–5.63)	5 (31.3)	2.06 (0.71–5.98)
**Household characteristics**					
Per capita household income (US$/day)	0.8 (0.3–1.3)	0.4 (0.1–1.0)	0.50 (0.28–0.89)	0.4 (0.1–1.0)	0.45 (0.19–1.09)
Flooding	184.0 (12.0)	4.0 (34.3)	0.95 (0.35–2.71)	5.0 (31.3)	3.35 (1.15–9.75)
Distance of residence:					
From the lowest point in valley (m)	23 (12–35)	15.0 (6.0–30.0)	0.97 (0.95–1.00)	17.5 (4.5–22.5)	0.96 (0.92–1.00)
To an open sewer (m)	19.8 (8.8–39.8)	21.6 (7.8–31.1)	1.00 (0.98–1.01)	6.9 (4.1–18.1)	0.95 (0.90–0.99)
**Peridomiciliary exposures**					
Cleaned clogged sewer	318 (20.7)	12 (34.3)	2.00 (0.98–4.05)	8 (50.0)	3.82 (1.42–10.27)
Contact with:					
Flood water	606 (39.6)	21 (60.0)	2.30 (1.16–4.55)	9 (56.3)	1.96 (0.73–5.30)
Sewage water	401 (26.1)	15 (42.9)	2.12 (1.08–4.18)	8 (50.0)	2.83 (1.05–7.58)
Mud	541 (35.3)	19 (54.3)	2.18 (1.11–4.27)	10 (62.5)	3.06 (1.11–8.46)
Garbage	330 (21.5)	12 (34.3)	1.90 (0.94–3.87)	7 (43.8)	2.84 (1.05–7.68)
**Workplace exposures**					
Refuse collection	4 (0.3)	0	0	1 (6.3)	18.56 (1.86–185.08)
Number of rats observed at workplace	3 (1.0–4.5)	5 (1.0–8.0)	1.08 (0.89–1.31)	12 (40–20.0)	1.27 (1.05–1.55)

Abbreviations: No., number; %, percentage; IQR, interquartile range; OR, odds ratio; CI, confidence interval.

In contrast to primary infection, we found that environmental attributes were significant risk factors for secondary infection in univariate analyses. Household flooding during rainy periods, proximity to open waste sewer, and three-dimensional distance of residence to the lowest point in the valley and open waste sewers had a stronger relationship with risk of secondary infection than primary infection. In contrast, risk behaviors that place participants in exposure to potentially contaminated environment near the household, such as contact with mud, sewage water, or garbage, and cleaning an open sewer, had a significant or near-significant association with both primary and secondary infections. Presence of rats at the place of residence, evaluated by the maximum number of rats seen, and sighting rats during the daytime, was not found as risk factor for primary nor secondary infection. Occupational factors, such as work that involved garbage collection and the maximum number of rats sighted at the workplace, were associated with increased risk for secondary infection in univariate analyses.

The multivariate multinomial model identified male gender (OR 2.88, 95% CI, 1.40–5.91) and per capita household income (OR 0.54 for an increase of $1 per person per day, 95% CI, 0.30–0.98) as independent risk factors for primary infection (Table 3). The model also identified age of 15–34 years (OR 10.82, 95% CI, 1.38–85.08) and proximity of the place of residence to the nearest open sewer (OR 0.95; 0.91–1.00 for an increase of 1 m distance) as risk factors for secondary infection. Contact with mud in the place of residence was found to have a non-significant association with both primary (OR 1.99, 95% CI 0.96–4.12) and secondary (OR 2.51, 95% CI 0.87–7.23) infection. Occupation-related exposures were not found to be significant risk factors for primary and secondary infection in the multivariate analyses.

**Table 3:** Multivariate risk factors for primary and secondary infection among cohort participants.

Factor	Primary infection n = 35		Secondary infection n = 16	
	OR	95% CI	OR	95% CI
Age group (years)				
5–14	1.00		1.00	
15–34	1.17	0.54–2.56	10.82	1.38–85.08
≥35	0.64	0.22–1.90	5.39	0.54–53.59
Male gender	2.88	1.40–5.91	2.33	0.84–6.41
Per capita household income (US$/day)	0.54	0.30–0.98	0.52	0.21–1.26
Proximity to an open sewer (m)	1.00	0.99–1.02	0.95	0.91–0.99
Contact with mud near the household	1.99	0.96–4.12	2.51	0.87–7.23

Abbreviations: OR, odds ratio; CI, confidence interval.

## Discussion

The findings of this large prospective investigation identified high rates of *Leptospira* infection among the study urban slum population, with more than 3% of the residents demonstrating serologic evidence of infection over a mean follow-up period of approximately one year. A single *L. interrogans* serogroup, serogroup Icterohaemorrhagiae, was the presumptive infecting agent, since highest agglutinating antibody titers were observed in 98% of participants with serologically-confirmed infection. Slum residents had an overall high risk for a repeat exposure and infection with the same agent. Furthermore, we identified that there are distinct risk factors for acquiring primary and secondary infection, suggesting that there exist sub-populations among slum residents who are repeatedly infected with leptospirosis.

These findings highlight the potential large and unrecognized burden of leptospirosis in urban slum settlements. There have been no comparable studies performed in slum settings that have followed large numbers of community-based participants and prospectively ascertained outcomes with standard serologic methods. A longitudinal study had been performed in Iquitos, Peru and found that annual incidence of *Leptospira* seroconversion was 288 per 1,000 persons based on IgM ELISA seroconversion during follow-up of 158 urban slum residents [33]. The much higher incidence observed in Iquitos may be due to the use of the IgM ELISA rather than the standard MAT method [33], which is more specific in detecting exposure to pathogenic leptospires [1], [3]. Alternatively, the difference may reflect differences in the frequency of infection among slum settlements that have distinct ecological and socio-economic characteristics.

A key knowledge gap in leptospirosis centers on the natural history of the disease and specifically, the proportion of infections that progress to develop disease and severe outcomes in the setting of high endemic transmission [1], [3]. Leptospirosis cases were not identified among the sample of 2,003 participants who participated in the cohort study to obtain direct estimates of the infection-to-severe-disease ratio. However, active hospital-based surveillance of the 12,651 community members that were identified during the baseline census found that the annual incidence of severe leptospirosis was 19.8 cases per 100,000 population at the study site during the cohort follow-up period. Comparison of this estimate of the severe disease incidence and the infection rate among cohort participants suggests that the infection-to-disease ratio may be as high as 191∶1 (95% CI, 82–542∶1). Although cohort participants with documented seroconversion did not report being hospitalized or visiting an ambulatory clinic for leptospirosis during follow-up, it is plausible that a significant proportion developed sub-clinical illness or clinical disease, which would not be identified and diagnosed as leptospirosis unless cases developed classic severe manifestations [1]. The high infection-to-disease ratio suggests that like dengue and other causes of acute fever in tropical urban environments, the burden of leptospirosis is under-recognized and significantly greater than reflected by reporting of severe cases.

The study findings indicate that the same *L. interrogans* serovar causes asymptomatic and sub-clinical infections as well as severe disease in this urban slum population. Among cohort participants with documented seroconversion, 98% had highest MAT titers that were directed against *L. interrogans* serovar Copenhageni, indicating that the Icterohaemorrhagiae serogroup was the infecting agent. We have observed that during long-term hospital-based surveillance in Salvador [4], [34], [35] and the study site community, >95% of the severe leptospirosis cases had highest MAT titers directed exclusively against the same serovar. *L. interrogans* serovar Copenhageni has been the sole serovar from serogroup Icterohaemorrhagiae to be isolated from this patient population during long-term surveillance [4], [7], as well as rat populations from the study community [36] and the city of Salvador [10], [37]. Together these findings indicate that transmission of leptospirosis is due to a single circulating serovar and provides additional evidence that *Rattus norvegicus*, the most common host of *L. interrogans* serovar Copenhageni [3], [10], is the principal reservoir in this urban slum setting. Moreover, our findings raise an important question with respect to what specific factors influence disease progression and the diverse range of clinical outcomes after infection with a single serovar agent. These factors may relate to strain-specific differences within the serovar that contribute to the strain's virulence, or alternatively, host-specific susceptibility or resistance factors and types of environmental exposures that contribute to the inoculum dose during infection [1], [3].

Re-infection was a frequent event among cohort participants during follow-up and raises the issue of whether natural infection confers immunity to a subsequent infection with a homologous serovar. Although there is clear evidence that immunization with live attenuated [38] and killed leptospires protects experimental animals against lethal infection [3], naturally-acquired immunity to re-infection in humans is poorly understood due to the limited number of prospective studies in well-characterized populations. A study from rural Andaman Islands used similar serologic criteria as employed in this study to prospectively identify *Leptospira* infection among school children and found that that primary infection rates were higher than secondary re-infection rates [39]. Additionally, the study observed a non-significant association between increased morbidity during follow-up and seronegative status during the baseline survey [39]. Although those findings are suggestive that a previous infection may protect against a subsequent infection, the conclusions were limited by the small numbers of clinical cases identified and potential confounding due to multiple circulating serovars.

Our study found that in an urban setting of transmission of a single serovar agent, prior exposure and infection did not confer complete protection against a subsequent serologically-ascertained, asymptomatic or sub-clinical infection with the same serovar. However, we could not determine whether prior infection protects an individual against developing clinical disease during subsequent re-infection since leptospirosis cases were not identified among cohort participants, nor could we evaluate the temporal relationship between initial infection and subsequent re-infection events due to the short follow-up period. Further prospective investigation is therefore needed to elucidate this question, which has major implications for development of an effective vaccine for leptospirosis in humans.

Urban slum residents who acquired a primary and secondary infection had some similar risk associations in common, yet also had important differences in infection rates and type of risk factors. Male gender and low socioeconomic status were independent risk factors for primary and secondary infections, although the associations with secondary infection were non-significant. Although male gender has not been identified as a risk factor for *Leptospira* infection in cohort studies performed in rural or mixed settings [33], [40], [41], males have a significantly higher risk for leptospirosis and anti-*Leptospira* antibodies in population-based surveillance [4], [23], [24] and seroprevalence surveys [9], [30], respectively, in Salvador and other urban settings [42]–[44]. We found that for each one-dollar increase in the daily per capita household income, the odds of primary and secondary infection decreased by 46% and 48%, respectively. Poverty and low socioeconomic status may contribute to infection risk through diverse mechanisms that include psychosocial processes that promote risky behaviors and exposures with a contaminated environment, the limited use of protective clothing against abrasions that facilitate entry of the *Leptospira* spirochete [21], lack of access to amenities and social support [45] and inadequate household sanitation conditions. A previous study in the same area demonstrated that adolescents and individuals who did not complete primary school had lower levels of knowledge and practices regarding leptospirosis [46]. Our prospective study confirms the findings of a seroprevalence survey performed in the same community [9] that relatively small differences in socioeconomic level, independent of poor environment, influence the risk for leptospirosis in slum populations characterized by overall high levels of absolute poverty.

The secondary infection rate was more than twice that of the primary infection rate (RR: 2.31; 95% CI: 1.30–4.10) among cohort participants, indicating that seropositive status at the baseline survey was a marker for increased risk for infection. In contrast to primary infection, individuals who had 15–34 years of age had significantly higher risk for acquiring a secondary infection (Table 3 and Figure 1), suggesting that a sub-population of young adults are repeatedly infected with pathogenic *Leptospira*. Furthermore we found that residence in proximity of an open sewer was significantly associated with an increased risk of primary infection and not secondary infection. It seems plausible that open sewers are a risk factor for primary infection, but the magnitude of this risk association was lower than that for secondary infection and not detected in this study. Together these finding suggest that there are distinct environmental settings and behavioral factors that contribute to repeat exposures.

Seasonal flooding is a frequent occurrence in slum communities in Salvador and especially for households situated on the poorest land quality at the bottom of valleys. Ganoza and colleagues found that environmental surface water from urban slums in Peru contained high concentrations of *L. interrogans* serovar Icterohaemorrhagiae [47]. Previous cross-sectional and case-control studies found that residence in flood-prone areas and in proximity to open sewer and rainwater drainage systems were associated with increased risk for anti-*Leptospira* antibodies and leptospirosis [9], [12], [23], [26]. The findings of this prospective study suggest that these infrastructure deficiencies of slum settlements also serve as transmission sources for repeated exposures to the *Leptospira* pathogen. Furthermore the findings demonstrate that adolescents and young adults are the primary risk group for repeated exposures and re-infection and indicate the need for specific interventions that target this high risk group.

This study has several limitations which need to be considered. The proportion of females and younger participants among study participants was greater than among non-participants. Infection rates that were adjusted for the age and gender distribution of the eligible population did not differ from crude rates, indicating that differences between enrolled and non-enrolled participants may have not introduced a significant bias in rate estimates. Among enrolled participants, 21% did not complete follow up due primarily to out-migration. This sub-group had a higher proportion of males, was better educated and had a higher income in comparison to those who completed follow up. Although we could not fully address the potential for bias, predictive factors for loss-to-follow-up were included in our modeling approach and the estimates for the risk associations may therefore be valid approximations.

In addition, our findings may not be broadly generalizable since the study was performed in a single slum community in Brazil. The incidence of *Leptospira* infection and risk associations identified in our study is expected to vary given the differences in underlying conditions of social deprivation, environmental degradation and climate where urban slum communities are situated. However the study site is a typical slum community in the city of Salvador and Brazil, where respectively, 33% [29] and 28% [5] of the population inhabit in such settlements. Furthermore, a large proportion of the one billion inhabitants of urban slums, as defined by the UN-HABITAT [5], reside in communities that have the same features of poverty, climate, poor environment and inadequate access to sanitation as found in our study site. The findings of this study may therefore be highly relevant to the situation of leptospirosis in urban slum settlements in developing countries and across tropical regions.

Furthermore, the study offers insights on the approaches needed to effectively address this neglected disease as the world's population of slum dwellers doubles to two billion by 2020 [48]. The study provides the first prospective evidence to support the assertion that defined infrastructure deficiencies in slum communities serve as transmission sources for leptospirosis. Removal of these sources, through implementation of adequate closed sewage and drainage systems, should therefore be a public health priority. Our findings also highlight the importance of adolescents and young adults as a risk group for spill-over infections. Efforts need to be made to identify and target through intervention the risky behaviors in this age group that promote recurring exposures with environmental contamination. Similarly, further work is required to identify the processes by which the social gradient of status influences unequal health outcomes within slum populations living in absolute poverty. By elucidating such mechanisms, we may not only identify effective prevention for leptospirosis, but may also identify common processes and interventions for the large range of communicable and non-communicable diseases that affect marginalized urban communities.

## Supporting Information

Checklist S1 STROBE checklist.
(DOC)

Table S1 Crude *Leptospira* infection rates among 1,585 participants of the cohort, according to age and gender.
(DOCX)
