# Supplementary material for: Prospective Study of Leptospirosis Transmission in an Urban Slum Community: Role of Poor Environment in Repeated Exposures to the Leptospira Agent
Source: PLoS Negl Trop Dis. 2014 May 29;8(5):e2927. doi: 10.1371/journal.pntd.0002927 (PMC4038618; doi:10.1371/journal.pntd.0002927)
Supplement: Table S1 — Crude Leptospira infection rates among 1,585 participants of the cohort, according to age and gender. (DOCX) [file pntd.0002927.s002.docx]

| **Type** |  | **Total** | | |  |  | **Male** | | |  |  | **Female** | | |  | **RR^b^** | **95% CI** | |  |
| --- | --- | --- | --- | --- | --- | --- | --- | --- | --- | --- | --- | --- | --- | --- | --- | --- | --- | --- | --- |
|  | No. cases (PY) | | Rate^a^ | 95% CI | | No. cases (PY) | | Rate^a^ | 95% CI | | No. cases (PY) | | Rate^a^ | 95% CI | |  |  |  |  |
| All infections | 51 (1,349) | | 37.8 | 26.3 – 51.9 | | 31 (569) | | 54.5 | 33.8 – 81.4 | | 20 (780) | | 25.6 | 13.9 – 41.9 | | **2.12** | **1.22 – 3.69** | |  |
| 5-14 years | 12 (368) | | 32.6 | 14.3 – 60.6 | | 9 (188) | | 47.9 | 17.8 – 96.9 | | 3 (180) | | 16.7 | 0.0 – 67.5 | | 2.87 | 0.79 -10.44 | |  |
| 15-24 years | 17 (355) | | 47.9 | 24.5 – 81.3 | | 12 (143) | | 83.9 | 37.6 – 156.0 | | 5 (212) | | 23.6 | 5.3 – 58.8 | | **3.55** | **1.28 – 9.88** | |  |
| 25-34 years | 14 (241) | | 58.1 | 27.4 – 103.6 | | 7 (97) | | 72.2 | 22.3 – 158.8 | | 7 (144) | | 48.6 | 15.5 – 106.9 | | 1.48 | 0.54 – 4.10 | |  |
| 35-44 years | 7 (187) | | 37.4 | 11.6 – 82.4 | | 2 (69) | | 29.0 | 0.0 – 110.9 | | 5 (118) | | 42.4 | 9.4 – 105.6 | | 0.70 | 0.14 – 3.53 | |  |
| >44 years | 1 (198) | | 5.05 | 0.0 – 29.4 | | 1 (72) | | 13.9 | 0.0 – 80.9 | | 0 (126) | | 0 | 0.0 – 29.2 | | --- | ----- | |  |
| Primary infections^c^ | 35 (1,126) | | 31.1 | 19.9 – 45.4 | | 22 (462) | | 47.6 | 26.7 – 76.2 | | 13 (664) | | 19.6 | 8.9 – 35.6 | | **2.43** | **1.24 – 4.78** | |  |
| 5-14 years | 12 (334) | | 35.9 | 15.7 – 66.8 | | 9 (168) | | 53.6 | 19.9 – 108.5 | | 3 (166) | | 18.7 | 15.4 – 56.2 | | 2.96 | 0.82 – 10.75 | |  |
| 15-24 years | 11 (292) | | 37.7 | 15.8 – 71.8 | | 8 (113) | | 70.8 | 24.3 – 148.9 | | 3 (179) | | 16.8 | 14.3 – 52.2 | | **4.22** | **1.14 – 15.59** | |  |
| 25-34 years | 7 (188) | | 37.2 | 11.5 – 81.9 | | 4 (71) | | 56.3 | 9.2 – 153.9 | | 3 (117) | | 16.9 | 14.4 – 52.7 | | 2.20 | 0.50 – 9.53 | |  |
| 35-44 years | 5 (156) | | 32.1 | 7.1 – 79.9 | | 1 (51) | | 19.6 | 0.0 – 114.2 | | 4 (105) | | 38.1 | 6.2 – 104.1 | | 0.51 | 0.06 – 4.48 | |  |
| >44 years | 0 (156) | | 0 | 0.0 – 23.6 | | 0 (59) | | 0 | 0.0 – 62.3 | | 0 (97) | | 0 | 0.0 – 38.0 | | --- | | ----- | |
| Secondary infections^d^ | 16 (223) | | 71.7 | 35.8 – 123.6 | | 9 (107) | | 84.1 | 31.2 – 170.3 | | 7 (116) | | 60.3 | 18.7 – 132.8 | | 1.39 | | 0.54 – 3.61 | |
| 5-14 years | 0 (34) | | 0 | 0.0 – 108.1 | | 0 (20) | | 0 | 0.0 – 183.8 | | 0 (14) | | 0 | 0.0 – 262.5 | | --- | | ----- | |
| 15-24 years | 6 (63) | | 95.2 | 25.8 – 221.4 | | 4 (30) | | 133.3 | 21.7 – 36.4 | | 2 (33) | | 60.6 | 0.0 – 231.9 | | 2.20 | | 0.43 – 11.16 | |
| 25-34 years | 7 (52) | | 134.6 | 41.7 – 296.2 | | 3 (25) | | 120.0 | 10.2 – 373.5 | | 4 (27) | | 148.1 | 24.1– 404.8 | | 0.81 | | 0.20 – 3.27 | |
| 35-44 years | 2 (31) | | 64.5 | 0.0 – 246.9 | | 1 (17) | | 58.8 | 0.0 – 342.6 | | 1 (14) | | 71.4 | 0 – 416.0 | | 0.82 | | 0.06 – 12.01 | |
| >44 years | 1 (43) | | 23.3 | 0.0 – 135.4 | | 1 (15) | | 66.7 | 0.0 – 388.3 | | 0 (28) | | 0 | 0.0 – 131.2 | | --- | | ----- | |

Abbreviations: PY: person-years of follow-up; RR: Risk ratio; CI: confidence intervals adjusted according to design effect and number of household clusters. Bold values indicate statistical significance.

^a^Rates expressed as infections per 1,000 person-years

^b^Risk ratio was calculated for males versus females

^c^Primary infection was defined as an increase in the microscopic agglutination test (MAT) titer for any of the tested serovars from zero in the first test to at least 50 in the second test.

^d^Secondary infection was defined as an increase in the MAT from zero in the first test to at least 50 in the second test, for someone who had had an initial titer ≥25 for a different serovar in the first test; or as an increase of four-fold in the MAT for the same serovar from an initial titer ≥25.
